# Supplementary material for: Management of Anastomotic Leakage after Colorectal Resection: Survey among the German CHIR-Net Centers
Source: J Clin Med. 2023 Jul 27;12(15):4933. doi: 10.3390/jcm12154933 (PMC10419945; doi:10.3390/jcm12154933)
Supplement: Supplementary file 1 [file jcm-12-04933-s001.zip › jcm-2511871-supplementary.pdf]

Supplementary files:

Table S1a-d, Table S2 and Table S3

Table S1a. Overview of the questions 1 to 4: center characteristics

Nr.	Question / choices	Response rate
1	Please state the level of care of your hospital	
	primary	7 (31.8)
	secondary	5 (22.7)
	tertiary	10 (45.5)
	academic	9 (90)
	non-academic	1 (10)
2	Please state the certified status of your department*	
	German Cancer Society (DKG)	
	colorectal center	8 (36.4)
	oncological center	10 (45.5)
	German Society of General and Visceral Surgery (DGAV)	
	colorectal center	6 (27.3)
	center for minimally invasive surgery	5 (22.7)
	n.a.	3 (13.6)
3	Does your department participate in the documentation in registers of DGAV-StudoQ?*	
	module for colon cancer	9 (40.9)
	module for rectal cancer	10 (45.5)
	module for diverticulitis	5 (22.7)
	no	9 (40.9)
4	How many colorectal resections with primary anastomosis are performed on a yearly average in your department?	
	annually median	150 [100-275]
	of which, colonic resections for cancer	70 [50-110]
	of which, rectal resections for cancer	40 [27.5-65]
	n.a.	1

StudoQ – national registry for quality management, n.a. no answer, * multiple selections possible. Data is presented as n (%) or median [IQR]

Table S1b. Overview of the questions 5 to 6: perioperative management

Nr.	Question / choices	Response rate
5	Do you apply a preoperative antegrade MBP?	
	yes	16 (72.3)
	no	5 (23.81)
	n.a.	1 (4.6)
5a	If yes, in which case?	
	resection with anastomosis and protective ostomy	5 (31.3)
	resection with anastomosis and without protective ostomy	0
	both	11 (68.8)
6	Which kind of bowel preparation do you apply?	
	MBP+/ABX-	1 (6.3)
	MBP+/ i.v. ABX+	1 (6.3)
	MBP +/ oral ABX+	8 (50.0)
	MBP+/ i.v. and oral ABX+	6 (37.5)

ABX anitbiotics; i.v. intravenous; MBP mechanical bowel preparation; n.a. no answer

Table S1c. overview of the questions 7 to 11: diagnostic of the colorectal anastomotic leakage

Nr.	Question / choices	Response rate
7	How many CAL are treated on a yearly average (e.g., 2018) in your department?	
	< 10	13 (59.1)
	11 – 20	6 (27.3)
	21 – 30	1 (4.6)
	n.a.	2 (9.1)
8	Which of the following diagnostic methods do you apply upon suspicion of CAL?*	
	flexible endoscopy	16 (72.7)
	computer tomography with contrast enema	19 (86.4)
	other	2 (9.1)
	n.a.	2 (9.1)
9	Who is performing the endoscopy in case of CAL suspicion?	
	only surgeons	2 (9.1)
	only gastroenterologists	4 (18.2)
	both	14 (63.6)
	n.a.	2 (9.1)
10	Would you consider an algorithm on the management of CAL to be better than a purely individual case-by-case decision regarding its treatment?	
	yes	17 (77.3)
	no	3 (13.6)
	n.a.	2 (9.1)
11	Do you apply the ISREC grading in the management of CAL?	
	yes	11 (50.0)
	no	9 (40.9)
	n.a.	2 (9.1)

CAL colorectal anastomotic leakage, ISREC International Study Group of Rectal Cancer, * multiple selections possible

Table S1d. Overview of the questions 12 to 27: management of the colorectal anastomotic leakage

Nr.	Question / choices	Response rate
12	In case of CAL, which kind of deviating ostomy would you prefer?	
	ileostomy	18 (81.8)
	colostomy	2 (9.1)
	n.a.	2 (9.1)
13	Should a leaking intraperitoneal anastomosis in an ASA 1–2 and < 80-year patient be preserved?	
	yes	16 (72.7)
	no	3 (13.6)
	n.a.	3 (13.6)
14	Should a leaking intraperitoneal anastomosis in an ASA ≥ 3 and/or ≥ 80-year patient be preserved?	
	yes	8 (36.4)
	no	11 (50.0)
	n.a.	3 (13.6)
15	Should a leaking extraperitoneal anastomosis in an ASA 1–2 and < 80-year patient be preserved?	
	yes	18 (81.8)
	no	1 (4.6)
	n.a.	3 (13.6)
16	Should a leaking extraperitoneal anastomosis in an ASA ≥3 and/or ≥ 80-year patient be preserved?	
	yes	13 (59.1)
	no	6 (27.3)
	n.a.	3 (13.6)
17	Do you apply ENPT for the treatment of CAL?	
	yes	18 (81.8)
	no	2 (9.1)
	n.a.	2
17a	if yes, up to what degree of dehiscence would you consider it?	
	up to ¼ of the circumference	1 (5.6)
	up to ½ of the circumference	9 (50.0)
	up to ¾ of the circumference	5 (27.8)
	entire circumference	3 (16.7)
18	Do you apply any techniques of transanal anastomotic repair for extraperitoneal CAL?	
	yes	12 (54.6)
	no	8 (36.4)
	n.a.	2 (9.1)
18a	if **yes**, how is the procedure performed for anastomoses	
	higher than 6 cm ab ano?	
	endoscopically	3 (25.0)
	open surgical approach	3 (25.0)
	not applicable	6 (50.0)
	up to 6 cm ab ano?	
	endoscopically	3 (25.0)
	open surgical approach	8 (66.7)
	n.a.	1 (8.3)
20	CAL grade B, non-ischemic bowel and ASA 1-2 and < 80-year patient after rectal resection **without** a primary deviating ostomy. Choose a treatment:	
	percutaneous drainage of the abscess	1 (4.6)
	endoscopic wash-out, with or without drainage placement	1 (4.6)
	endoscopic wash-out and ENPT	9 (40.9)
	same as a CAL grade C	9 (40.9)
	n.a.	2 (9.1)
20a	Would you treat the previous patient but in case of poor vascularization at the site of the anastomosis as a CAL grade C	

	yes	19 (86.4)
	no	1 (4.6)
	n.a.	2 (9.1)
21	CAL grade B, non-ischemic bowel and ASA ≥ 3 and/or ≥ 80-year patient after rectal resection **without** a primary deviating ostomy. Choose a treatment:	
	percutaneous drainage of the abscess	0
	endoscopic wash-out, with or without drainage placement	0
	endoscopic wash-out and ENPT	7 (31.8)
	same management as a CAL grade C	13 (59.1)
	n.a.	2 (9.1)
21a	Would you treat the previous patient but in case of poor vascularization at the site of the anastomosis as a CAL grade C?	
	yes	19 (86.4)
	no	1(4.6)
	n.a.	2 (9.1)
22	CAL grade C, non-ischemic bowel and ASA 1-2 and < 80-year patient after rectal resection **without** a primary deviating ostomy. Choose a treatment	
	endoscopic wash-out, ENPT and deviating ileostomy	7 (31.8)
	surgery, drainage of the abscess and deviating ileostomy	2 (9.1)
	surgery, drainage of the abscess, repair of the anastomosis	1 (4.6)
	surgery, drainage of the abscess, repair of the anastomosis and deviating ileostomy	10 (45.5)
	surgery, drainage of the abscess, breakdown of the anastomosis and permanent colostomy.	0
	n.a.	2 (9.1)
22a	How would you treat the previous patient in case of poor vascularization at the site of the anastomosis?	
	endoscopic wash-out, ENPT and deviating ileostomy	2 (9.1)
	surgery, drainage of the abscess and deviating ileostomy	1 (4.6)
	surgery, drainage of the abscess, repair of the anastomosis	0
	surgery, drainage of the abscess, repair of the anastomosis and deviating ileostomy	4 (18.2)
	surgery, drainage of the abscess, breakdown of the anastomosis and permanent colostomy.	13 (59.1)
	n.a.	2 (9.1)
23	CAL grade C, non-ischemic bowel and ASA ≥ 3 and/or ≥ 80-year patient after rectal resection **without** a primary deviating ostomy. Choose a treatment:	
	endoscopic wash-out, ENPT and deviating ileostomy	7 (31.8)
	surgery, drainage of the abscess and deviating ileostomy	2 (9.1)
	surgery, drainage of the abscess, repair/redo of the anastomosis	1 (4.6)
	surgery, drainage of the abscess, repair of the anastomosis and deviating ileostomy	5 (22.7)
	surgery, drainage of the abscess, breakdown of the anastomosis and permanent colostomy.	5 (22.7)
	n.a.	2 (9.1)
23a	How would you treat the previous patient in case of poor vascularization at the site of the anastomosis?	
	endoscopic wash-out, ENPT and deviating ileostomy	1 (4.6)
	surgery, drainage of the abscess and deviating ileostomy	0
	surgery, drainage of the abscess, repair/redo of the anastomosis	0
	surgery, drainage of the abscess, repair of the anastomosis and deviating ileostomy	3 (13.6)
	surgery, drainage of the abscess, breakdown of the anastomosis and permanent colostomy.	16 (72.7)
	n.a.	2 (9.1)
24	CAL grade B and ASA 1-2 and < 80-year patient after rectal resection **with** a primary deviating ostomy. Choose a treatment:	
	percutaneous drainage of the abscess	3 (13.6)
	endoscopic wash-out, with or without drainage placement	2 (9.1)
	endoscopic evacuation and ENPT	15 (68.2)
	same as a CAL grade C	0
	n.a.	2 (9.1)

24a	Would you treat the previous patient but in case of poor vascularization at the site of the anastomosis as a CAL grade C	
	yes	17 (77.3)
	no	3 (13.6)
	n.a.	2 (9.1)
25	CAL grade B and ASA ≥ 3 and/or ≥ 80-year patient after rectal resection **with** a primary deviating ostomy. Choose a treatment:	
	percutaneous drainage of the abscess	3 (13.6)
	endoscopic wash-out, with or without drainage placement	4 (18.2)
	endoscopic evacuation and ENPT	13 (59.1)
	same management as a CAL grade C	0
	n.a.	2 (9.1)
25a	Would you treat the previous patient but in case of poor vascularization at the site of the anastomosis as a CAL grade C?	
	yes	17 (77.3)
	no	3 (13.6)
	n.a.	2 (9.1)
26	CAL grade C, non-ischemic bowel, ASA 1-2 and < 80-year patient after LAR **with** a primary deviating ostomy. Choose a treatment:	
	Surgery, lavage and drainage of the abscess	5 (22.7)
	Surgery, drainage of the abscess, repair/redo of the anastomosis	13 (59.1)
	Surgery, drainage of the abscess, breakdown of the anastomosis and permanent colostomy	1 (4.6)
	n.a.	3 (13.6)
26a	How would you treat the previous patient but in case of poor vascularization at the site of the anastomosis?	
	Surgery, lavage and drainage of the abscess	0
	Surgery, drainage of the abscess, repair/redo of the anastomosis	10 (45.5)
	Surgery, drainage of the abscess, breakdown of the anastomosis and permanent colostomy	10 (45.5)
	n.a.	2 (9.1)
27	CAL grade C, non-ischemic bowel and ASA ≥ 3 and/or ≥ 80-year patient after rectal resection **with** a primary deviating ostomy. Choose a treatment	
	Surgery, lavage and drainage of the abscess	4 (18.2)
	Surgery, drainage of the abscess, repair/redo of the anastomosis	10 (45.5)
	Surgery, drainage of the abscess, breakdown of the anastomosis and permanent colostomy	6 (27.3)
	n.a.	2 (9.1)
27a	How would you treat the previous patient but in case of poor vascularization at the site of the anastomosis?	
	Surgery, lavage and drainage of the abscess	0
	Surgery, drainage of the abscess, repair/redo of the anastomosis	2 (9.1)
	Surgery, drainage of the abscess, breakdown of the anastomosis and permanent colostomy	18 (81.8)
	n.a.	2 (9.1)

ASA American Society of Anesthesiologists, CAL colorectal anastomotic leakage, ENPT endoscopic negative pressure therapy, surgery re-laparotomy or re-laparoscopy, n.a. no answer

Table S2. Remarks to the survey

1.	ASA and age are not enough criteria for decision-making which is usually patient tailored
2.	The questions should have included additional specification about patient condition (e.g., in case of sepsis a Hartmann's situation would probably be preferred regardless ASA and age)
3.	Proper vascularization means a possibility to preserve the anastomosis, however it is important to start the therapy within 12 hours from diagnosis. On the other side, ischemic bowel means bad prognosis
4.	I couldn't answer all questions and in some cases was hard to stay objective because they were not flexible enough
5.	There is too little option for patient-tailored decision-making
6.	Few questions could not be answered only with yes or no

ASA American Association of Anesthesiologists

Table S3. Which is in your opinion the most important knowledge gap regarding the therapy of CAL?

1. The influence of the microbiome
2. Why does an anastomosis leak although technically everything was correct?
3. There is not enough evidence about the etiopathogenesis of the CAL. There is a lack of studies on ENPT with versus without protective ostomy in case of CAL
4. Close follow-up in order to act immediately. Prophylactic ENPT for low rectal carcinoma
5. When should an anastomosis be preserved in respect to mortality?
6. Patient tailored decision-making
7. The rate of stenosis and LARS after successful non-surgical therapy
8. There is scarce data and no RCTs on management of a leaking anastomosis. Is there a difference between colon and rectum anastomotic leakage? The preoperative fecal continence and the social aspects are seldom considered when decision-making

CAL colorectal anastomotic leakage, ENPT endoscopic negative pressure therapy, LARS low anterior resection syndrome, RCT randomized controlled trials
